# PubChem3D: Shape compatibility filtering using molecular shape quadrupoles

**DOI:** 10.1186/1758-2946-3-25

**Published:** 2011-07-20

**Authors:** Sunghwan Kim, Evan E Bolton, Stephen H Bryant

**Affiliations:** 1 National Center for Biotechnology Information, National Library of Medicine, National Institutes of Health, Department of Health and Human Services, 8600 Rockville Pike, Bethesda, MD 20894, USA

## Abstract

**Background:**

PubChem provides a 3-D neighboring relationship, which involves finding the maximal shape overlap between two static compound 3-D conformations, a computationally intensive step. It is highly desirable to avoid this overlap computation, especially if it can be determined with certainty that a conformer pair cannot meet the criteria to be a 3-D neighbor. As such, PubChem employs a series of pre-filters, based on the concept of volume, to remove approximately 65% of all conformer neighbor pairs prior to shape overlap optimization. Given that molecular volume, a somewhat vague concept, is rather effective, it leads one to wonder: can the existing PubChem 3-D neighboring relationship, which consists of billions of shape similar conformer pairs from tens of millions of unique small molecules, be used to identify additional shape descriptor relationships? Or, put more specifically, can one place an upper bound on shape similarity using other "fuzzy" shape-like concepts like length, width, and height?

**Results:**

Using a basis set of 4.18 billion 3-D neighbor pairs identified from single conformer per compound neighboring of 17.1 million molecules, shape descriptors were computed for all conformers. These steric shape descriptors included several forms of molecular volume and shape quadrupoles, which essentially embody the length, width, and height of a conformer. For a given 3-D neighbor conformer pair, the volume and each quadrupole component (Q_x_, Q_y_, and Q_z_) were binned and their frequency of occurrence was examined. Per molecular volume type, this effectively produced three different maps, one per quadrupole component (Q_x_, Q_y_, and Q_z_), of allowed values for the similarity metric, shape Tanimoto (ST) ≥ 0.8.

The efficiency of these relationships (in terms of true positive, true negative, false positive and false negative) as a function of ST threshold was determined in a test run of 13.2 billion conformer pairs not previously considered by the 3-D neighbor set. At an ST ≥ 0.8, a filtering efficiency of 40.4% of true negatives was achieved with only 32 false negatives out of 24 million true positives, when applying the separate Q_x_, Q_y_, and Q_z _maps in a series (Q_xyz_). This efficiency increased linearly as a function of ST threshold in the range 0.8-0.99. The Q_x _filter was consistently the most efficient followed by Q_y _and then by Q_z_. Use of a monopole volume showed the best overall performance, followed by the self-overlap volume and then by the analytic volume.

Application of the monopole-based Q_xyz _filter in a "real world" test of 3-D neighboring of 4,218 chemicals of biomedical interest against 26.1 million molecules in PubChem reduced the total CPU cost of neighboring by between 24-38% and, if used as the initial filter, removed from consideration 48.3% of all conformer pairs at almost negligible computational overhead.

**Conclusion:**

Basic shape descriptors, such as those embodied by size, length, width, and height, can be highly effective in identifying shape incompatible compound conformer pairs. When performing a 3-D search using a shape similarity cut-off, computation can be avoided by identifying conformer pairs that cannot meet the result criteria. Applying this methodology as a filter for PubChem 3-D neighboring computation, an improvement of 31% was realized, increasing the average conformer pair throughput from 154,000 to 202,000 per second per CPU core.

## Background

PubChem is an open and free resource of the biological activities of small molecules [1-4]. PubChem has an integrated theoretical 3-D layer, PubChem3D [5-7], which provides a precomputed 3-D neighboring relationship called "Similar Conformers" [7] to help users locate and relate data in the archive. "Similar Conformers" identifies chemicals with similar 3-D shape and similar 3-D orientation of functional groups typically used to define pharmacophores (defined here simply as "features"), complementing a PubChem 2-D neighboring relationship called "Similar Compounds", which identifies closely related chemical analogs using the PubChem 2-D subgraph fingerprint [8]. Effectively, for each PubChem chemical structure, this 3-D neighboring relationship provides (at the time of writing) the results of a 3-D similarity search against 28.9 million compound records using three diverse conformers per molecule.

The PubChem3D neighboring uses as a measure of molecular shape similarity the shape Tanimoto (ST) [9,10], given as the following equation:(1)

where *V_AA _*and *V_BB _*are the self-overlap volumes of conformers A and B, respectively, and *V_AB _*is the common overlap volume between A and B. The 3-D neighboring requires finding the maximum shape similarity between static compound 3-D conformations, as dictated by *V_AB _*in **Equation 1**, to calculate ST, a computationally intensive step. It is highly desirable to avoid this overlap computation, especially if it can be determined with certainty that a conformer pair cannot meet the criteria to be a 3-D neighbor. As such, PubChem employs a series of filters, based on the concept of volume, to effectively ignore approximately 65% of all conformer neighbor pairs during 3-D neighboring, thus dramatically accelerating processing [7].

Volume, although a rather fuzzy concept, is rather effective as a filter between conformers dissimilar in shape and features [7]. Conceivably there are other aspects of molecular shape beyond volume to "recognize" when two shapes are (dis)similar. A characteristic one can readily imagine are descriptors associated with aspects of length, width, and height. Steric shape quadrupoles embody such a concept and attempts have been made to use their differences as a shape similarity metric [11,12]. This leads to the question: can additional simple shape descriptor relationships be identified that improve upon the volume-based filtering efficacy? Or, put another way, can one place an upper bound on shape similarity by identification of some (additional) crude shape compatibility between conformers?

In this paper, we examine the use of shape descriptors as a means to rapidly identify "dissimilar" molecule shapes. As a part of this, we attempt to answer the critical questions: are vague shape descriptors representing the concepts of length, width, and height good discriminators of molecular shape? Can 3-D similarity searching speed be further accelerated using shape descriptors more sophisticated than volume? Is it possible to create a "shape compatibility" mapping indexed to shape similarity?

## Results and Discussion

### 1. Distribution of shape descriptor components and their volume dependency

**Figure 1:**
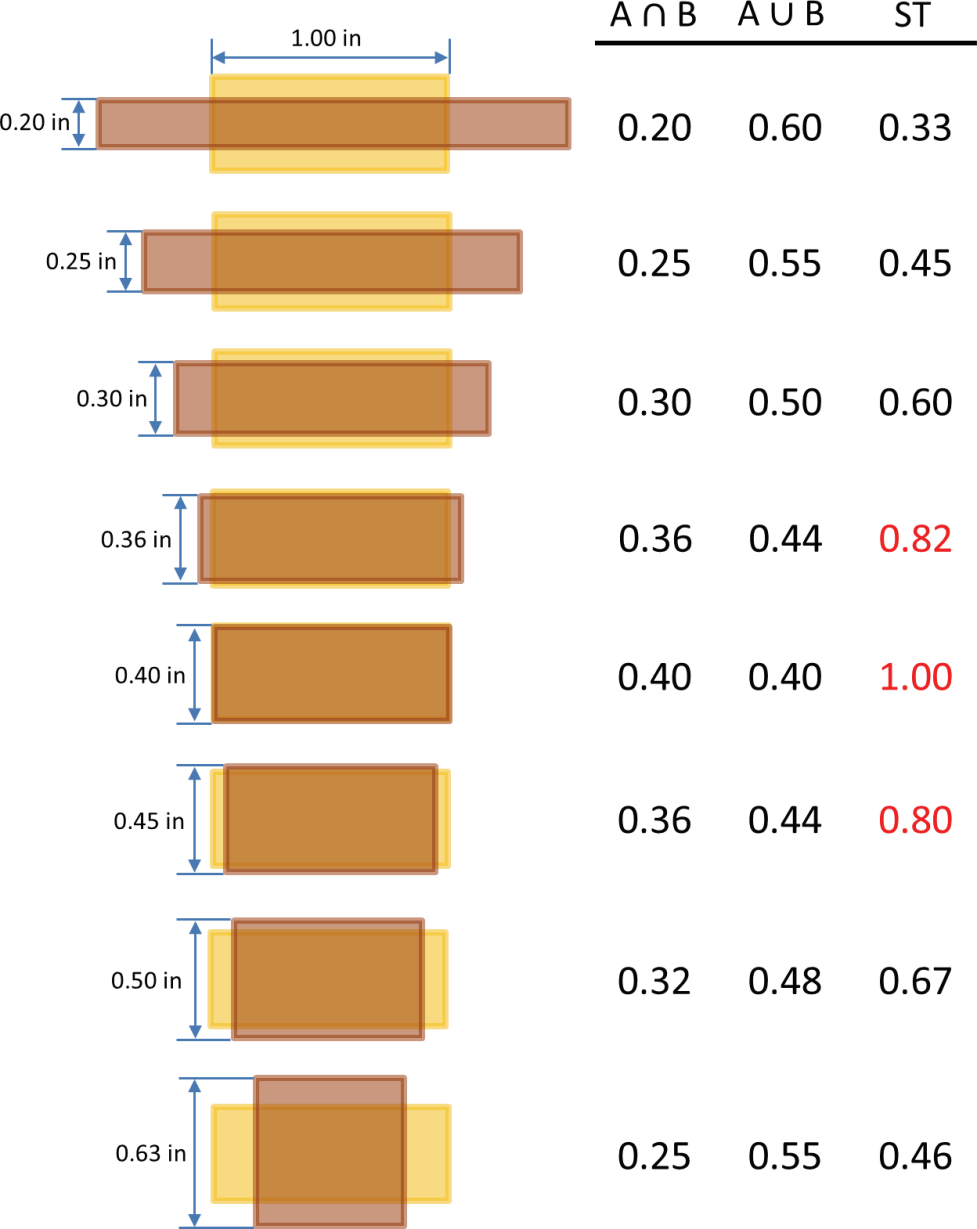
**Small changes in dimensions can result in large changes in overlap**. Using a 2-D rectangle shape with constant area (0.4 in^2^), one can see that small changes in shape dimensions (length and width) can result in large changes in shape overlap (ST). Note that, for two shapes to be considered similar to each other (with a ST score of ≥ 0.8, indicated in red), the difference in length and width between them should be smaller than a certain threshold.

The molecular shape quadrupoles in the principal-axes frame [9,13] are given as the following:(2)

where, *Q_x_, Q_y_*, and *Q_z _*are the x, y, and z components of the quadrupole moment, respectively. The x, y, and z components are conceptually equivalent to the length, width, and height of a molecule, respectively, with the largest quadrupole component defined as *Q_x _*and the smallest as *Q_z_*, by convention. An assumption underlying this study is that there is a point whereby, if the shape quadrupole difference between two conformers is too large, they cannot meet the ST ≥ 0.8 threshold required by PubChem3D neighboring, as illustrated in Figure 1. This relationship, if it actually exists, would allow conformer pairs to be filtered out, avoiding the time-consuming shape superposition optimization step for those pairs and enhancing the throughput of the PubChem 3-D neighboring. To attempt to determine if a relationship can be found, the shape quadrupole differences for known 3-D "Similar Conformers" were analyzed.

**Figure 2:**
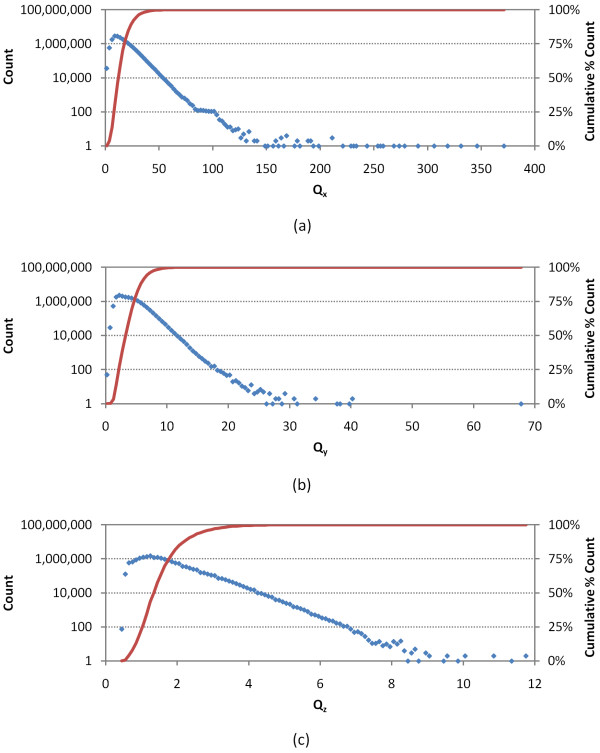
**Quadrupole distribution**. The frequency of occurance of the three quadrupole moment components for 17.1 million molecules from the PubChem Compound database, where (a) *Q_x_*, (b) *Q_y_*, and (c) *Q_z _*were binned into units of 2.5 Å^5^, 0.5 Å^5^, and 0.1 Å^5^, respectively.

**Figure 3:**
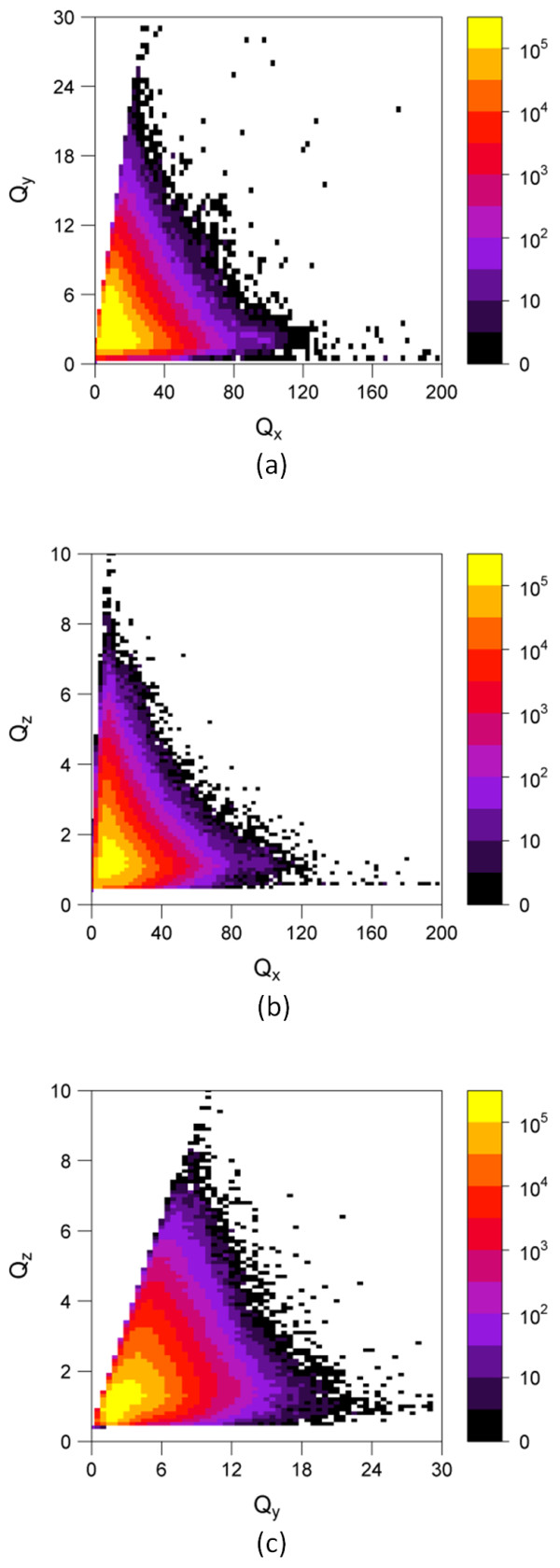
**Quadrupole interdependence**. The distribution of 17.1 million molecules from the PubChem Compound database as a function of (a) *Q_x _*and *Q_y_*, (b) *Q_x _*and *Q_z_*, and (c) *Q_y _*and *Q_z_*, respectively. *Q_x_, Q_y_*, and *Q_z _*were binned into units of 2.5 Å^5^, 0.5 Å^5^, and 0.1 Å^5^, respectively. The legend indicates the frequency of observation.

At the time of quadrupole filter project initiation (in October, 2008), 3-D neighboring of 17,143,181 unique molecules, effectively covering the CID range 1-25,000,000, had been completed using a single conformer per compound, yielding 4,182,412,802 3-D neighbors. Table 1 shows the statistics of the three quadrupole components for those 17.1 million molecules. The mean and standard deviation for *Q_x_, Q_y_*, and *Q_z _*were 15.01 ± 8.07 Å^5^, 3.81 ± 1.80 Å^5^, and 1.52 ± 0.65 Å^5^, respectively. Figure 2 and 3 display the distributions of *Q_x_, Q_y_*, and *Q_z_*, after they were binned into units of 2.5 Å^5^, 0.5 Å^5^, and 0.1 Å^5^, respectively. All three components showed strongly skewed distributions; however, most of the molecules were populated near the mean and relatively few molecules had quadrupole components much larger than the mean values.

**Table 1:** Quadrupole statistics

	Minimum	Median	Mean	Maximum	Standard Deviation
Q_x _	0.46	13.18	15.01	370.31	± 8.07
Q_y _	0.46	3.49	3.81	67.99	± 1.80
Q_z _	0.46	1.39	1.52	11.78	± 0.65

Distribution of the quadrupole moment components (Q_x_, Q_y_, and Q_z_), in units of Å^5^, for the 17.1 million molecules considered from the PubChem Compound database.

The molecular volume and quadrupole moments are correlated with each other according to the following equation:(3)

where *R_g _*is the radius of gyration and *V_mp _*is the monopole volume, which corresponds to the monopole in the shape multipole expansion [13]. **Equation 3 **implies that the size of a molecule (represented by the molecular volume) is not completely independent of its quadrupole moment. Therefore, at the beginning of this study, the correlation between molecular volume and quadrupole moment was investigated. Note that, because the molecular volume is not a measurable quantity with a clear, unanimous definition, there are many ways to estimate it [13-18]. Therefore, in addition to the monopole volume, the PubChem 3-D information includes two other volumes computed in different ways. One is the analytic volume and the other is the self-overlap volume. The analytic volume is considered to be most consistent to other definitions of molecular volume among the three, but its computation is also the slowest. For this reason, evaluation of the ST score given in **Equation 1 **uses the self-overlap volume, whose evaluation is considerably faster than the analytic volume; however, it typically overestimates the molecular volume by a factor of three greater than the analytic volume, as shown in Table 2. Each compound conformer record in the PubChem provides all three volumes and they can be downloaded: individually from the Compound Summary pages, using a list from the PubChem Download Facility (http://pubchem.ncbi.nlm.nih.gov/pc_fetch), or in bulk from the PubChem FTP site (ftp://ftp.ncbi.nlm.nih.gov/pubchem). To avoid confusion about these three different volumes used in the present paper, we denote the monopole volume, self-overlap volume, and analytic volume as *V*_mp_, *V*_so_, and *V*_an_, respectively, whereas the volume in a general sense is denoted as *V *(without any subscript).

**Table 2:** Volume statistics

	Minimum	Median	Mean	Maximum	Standard Deviation
*V_an _*	13.3	286.9	286.5	894.1	± 76.1
*V_mp_*	13.3	511.2	510.3	1,165.2	± 141.5
*V_so_*	12.8	1,082.9	1,081.9	2,517.6	± 315.9

Distribution of the analytic volume (*V_an_*), monopole volume (*V_mp_*), and self-overlap volume (*V_so_*), in units of Å^3^, for the 17.1 million molecules considered from the PubChem Compound database.

**Figure 4:**
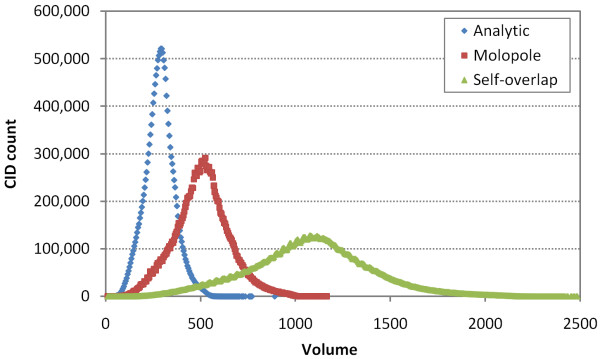
**Volume distribution**. The frequency of occurance of the three different volume types, analytic volume (*V_an_*, blue), monopole volume (*V_mp_*, red), and self-overlap volume (*V_so_*, green), for 17.1 million molecules from the PubChem Compound database, where all three volumes were binned into units of 5.0 Å^3^.

**Figure 5:**
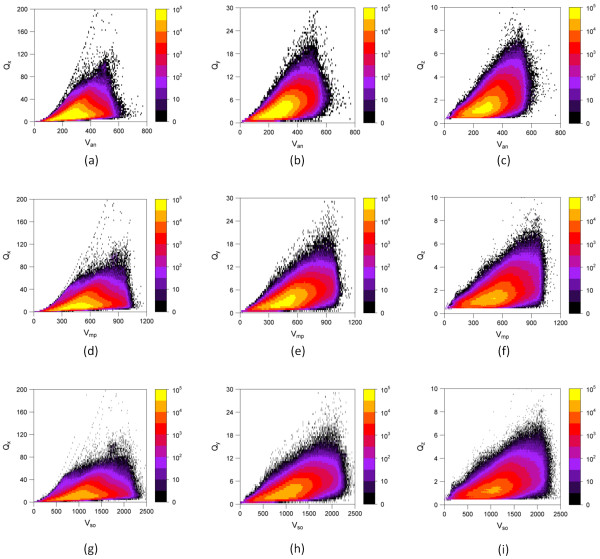
**Volume-quadrupole interdependence**. The distribution of 17.1 million molecules from the PubChem Compound database as a function of the molecular volume type and quadrupole component. *V_an _*[in panel (a)-(c)], *V_mp _*[in panel (d)-(f)], and *V_so _*[in panel (g)-(i)] indicate the analytic volume, monopole volume, and self-overlap volume, respectively. *Q_x _*[in panel (a), (d) and (g)], *Q_y _*[in panel (b), (e) and (h)], and *Q_z _*[in panel (c), (f) and (i)] indicate the three components of the quadrupole moment. All three volumes were binned into units of 5.0 Å^3 ^and the *Q_x_, Q_y_*, and *Q_z _*were binned into units of 2.5 Å^5^, 0.5 Å^5^, and 0.1 Å^5^, respectively. The legend indicates the frequency of observation.

Figure 4 displays the distribution of the three different volumes of the 17.1 million molecules from the PubChem Compound database. In general, *V*_so _is the largest, and *V*_an _is the smallest. As shown in Figure 5, the quadrupole moment increases with molecular size, implying that the effect of quadrupole difference between two molecules upon their shape similarity may depend on their relative molecular sizes. Therefore, the quadrupole differences of 3-D "Similar Conformer" neighbors as a function of volume need to be considered.

### 2. Design of 3-D neighbor filters using quadrupole moment differences

As a general premise, if two molecules with the same volume also have identical values for the quadrupole components, they are likely to be shape similar to each other. In addition, as the quadrupole moment difference deviates from zero, the maximum shape similarity is expected to decrease (see Figure 1). When the quadrupole (and volume) difference becomes greater than some value or threshold, the shape dissimilarity is such that the molecule conformer pair cannot possibly meet the criteria to be a PubChem 3-D neighbor (ST ≥ 0.8). Therefore, if we know these quadrupole difference thresholds for a given volume pair, one may be able to preclude conformer pairs that are not sufficiently shape similar, using only knowledge of the volume and quadrupole moments.

In the present study, the quadrupole moment differences of the 4.18 billion 3-D neighbors, identified from the 3-D neighboring of 17.1 million molecules, were analyzed to find the maximum possible quadrupole differences for two molecules to be neighbors (see also the "*Materials and Methods*" section). The volume and quadrupole moments of the two molecules in each neighbor pair were first converted into an integer value by using the following two equations:(4)(5)

where superscript "bin" is used to distinguish these integers from the original, non-binned values. The denominator *Binsize *was 5.0 Å^3 ^for all the three volumes, and 2.5 Å^5^, 0.5 Å^5^, and 0.1 Å^5^, for *Q_x_, Q_y_*, and *Q_z_*, respectively. After all 4.18 billion 3-D neighbors were binned according to their *V^bin ^*and *Q^bin ^*values, the 3-D neighbor distribution for a given (, ) pair was analyzed as a function of Δ*Q^bin^*.

**Figure 6:**
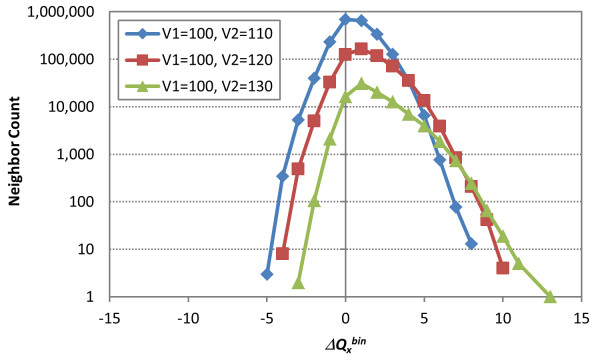
**Quadrupole difference tolerance**. The distributions of the 3-D neighbors as a function of the binned quadrupole differences, , of the two moleclues in each neighbor for (, ) = (100, 110), (100, 120), and (100, 130), respectively, illustrating how the frequency of Δ*Q^bin ^*rapidly decreases to zero as a function of magnitude.

To illustrate the general premise above that quadrupole deviations from zero result in a reduction is shape similarity, Figure 6 shows the neighbor count as a function of  for (, ) = (100, 110), (100, 120), and (100, 130). As anticipated, maximum neighbor populations exist when  is near the origin, and rapidly decrease in count (nearly linear reduction on a log curve) as the  deviates from zero. In addition, for a given () pair, neighbors were observed only for a certain range of , indicating that this range information can be used as a filter that pre-screens non-neighbor pairs. The asymmetric distribution of the 3-D neighbors in Figure 6 with respect to the ordinate axis () suggests that two different filters would need to be generated: one for  and the other for .

**Figure 7:**
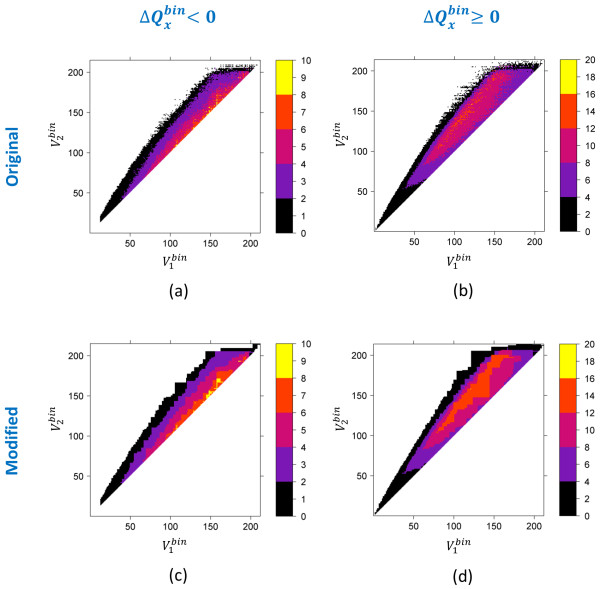
**Monopole volume  shape compatibility map and filter**. The absolute value of the maximum possible value of  for two molecules to be 3-D neighbors of each other, as a function of binned monopole volumes,  and  of molecules 1 and 2, respectively, at ST ≥ 0.8. The legend indicates the absolute value of .

**Figure 8:**
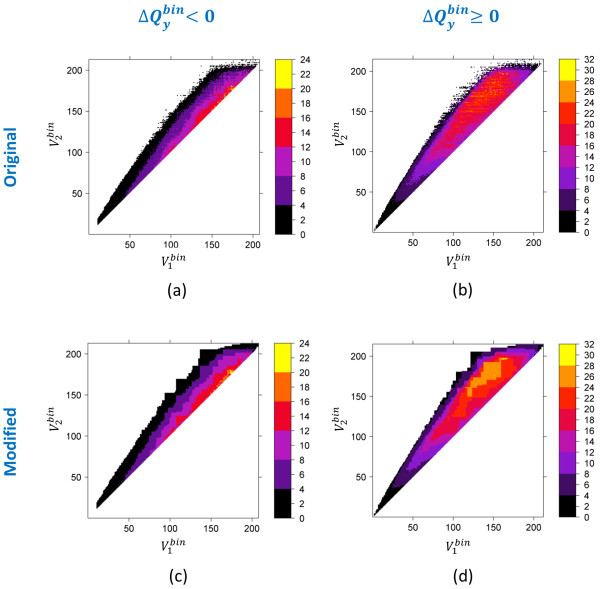
**Monopole volume  shape compatibility map and filter**. The absolute value of the maximum possible value of  for two molecules to be 3-D neighbors of each other, as a function of binned monopole volumes,  and  of molecules 1 and 2, respectively, at ST ≥ 0.8. The legend indicates the absolute value of .

**Figure 9:**
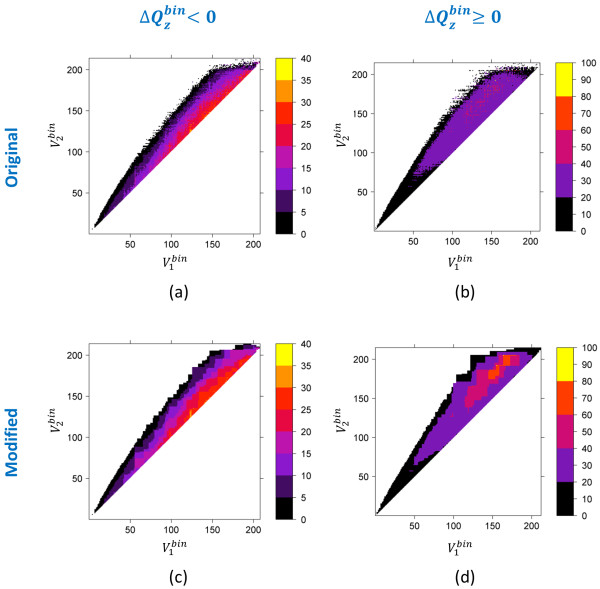
**Monopole volume  shape compatibility map and filter**. The absolute value of the maximum possible value of  for two molecules to be 3-D neighbors of each other, as a function of binned monopole volumes,  and  of molecules 1 and 2, respectively, at ST ≥ 0.8. The legend indicates the absolute value of

Figures 7, 8 and 9 show the Δ*Q^bin ^*threshold for each quadrupole component as a function of volume for the 4.18 billion 3-D neighbors. Note that, since PubChem regularly gets additional new unique content from its contributors, there is always a possibility that the 3-D neighboring of these new records may identify previously unseen cases of Δ*Q^bin ^*threshold. If we use these Δ*Q^bin ^*threshold maps [see panels (a) and (b) of Figures 7, 8 and 9] as a filter during neighboring, we would preclude those 3-D neighbors. Therefore, we modified the maps [see panels (c) and (d) of Figures 7, 8 and 9], as described in the "*Materials and Methods*" section, to extend Δ*Q^bin ^*difference values or to add neighboring bins where no population is found in an attempt to mitigate any such issues in the fringe regions on the maps.

These modified Δ*Q^bin ^*threshold maps are designated as quadrupole filters. For simplicity, we name these filters with a capital letter "**F**" followed by a subscript, which represents one of the quadrupole components, and a superscript, which represents the type of volume involved. For example, filter "" indicates that the *Q_x _*filter generated with the analytic volume, *V_an_*.

Given that these quadrupole filters were built using an existing set of 3-D neighbor cases, one needs to validate the extent of their efficacy. To do so, a 13.2 billion molecule conformer pair test set not considered as a part of the original 3-D neighboring training set, is utilized (see the "Materials and Methods" section). After computing the ST scores for the 13.2 billion pairs, the fraction of 3-D neighbors and non-neighbors, which would have been pre-screened if the quadrupole filters were applied, is summarized in Table 3.

**Table 3:** Accuracy of filtering as a function of volume type and quadrupole component at ST ≥ 0.80 threshold

	TP*^a^* (millions)	FP*^b^* (millions)	FN*^c^*	TN*^d^* (millions)
**Analytic Volume**				
Q_x_	24	9,855	3	3,290
	(0.2%)	(74.8%)	(0.0%)	(25.0%)
Q_y_	24	10,726	0	2,419
	(0.2%)	(81.4%)	(0.0%)	(18.4%)
Q_z_	24	11,036	1	2,108
	(0.2%)	(83.8%)	(0.0%)	(16.0%)
Q_xyz_	24	9,323	4	3,822
	(0.2%)	(70.8%)	(0.0%)	(29.0%)
**Monopole Volume**				
Q_x_	24	8,368	30	4,777
	(0.2%)	(63.5%)	(0.0%)	(36.3%)
Q_y_	24	9,224	2	3,921
	(0.2%)	(70.0%)	(0.0%)	(29.8%)
Q_z_	24	9,551	0	3,594
	(0.2%)	(72.5%)	(0.0%)	(27.3%)
Q_xyz_	24	7,818	32	5,327
	(0.2%)	(59.4%)	(0.0%)	(40.4%)
**Self-Overlap Volume**				
Q_x_	24	8,684	234	4,461
	(0.2%)	(65.9%)	(0.0%)	(33.9%)
Q_y_	24	9,223	56	3,922
	(0.2%)	(70.0%)	(0.0%)	(29.8%)
Q_z_	24	9,835	52	3,310
	(0.2%)	(74.7%)	(0.0%)	(25.1%)
Q_xyz_	24	7,993	288	5,152
	(0.2%)	(60.7%)	(0.0%)	(39.1%)

*^a ^*TP (true positive) = conformer pairs with ST≥0.8 that passed the quadrupole filter.

*^b ^*FP (false positive) = conformer pairs with ST<0.8 that passed the quadrupole filter.

*^c ^*FN (false negative) = conformer pairs with ST≥0.8 that were filtered out by the quadrupole filter.

*^d ^*TN (true negative) = conformer pairs with ST < 0.8 that were filtered out by quadrupole filter.

Of the three volume types utilized, the monopole-based quadrupole filters, **F***^mp^*, is arguably the best. Filter  removed 4.78 billion pairs (36.3%), while incurring a loss of only 30 out of 24 million "potential" neighbors. [Note that the definition of a PubChem 3-D neighbor involves *feature *similarity as well as *shape *similarity, while the quadrupole filters deal only with shape similarity. As such, the 30 pairs filtered out had a ST score sufficient to be a 3-D neighbor, making it a "potential" 3-D neighbor.] The false negative count of 30 removed by  is negligible, but does show that use of such a filter will result in precluding some potential 3-D neighbors in its use, in this case at a rate of 1 in 800,000.

Filters  and  are not as efficient as , but could still filter out 3.92 billion pairs (29.8%), and 3.59 billion pairs (27.3%), respectively, when considered individually. If the three **F***^mp ^*filters are used in a series (denoted as , and applied one after the other), 5.33 billion pairs (40.4%) could be removed with a loss of only 32 potential neighbors. Filter **F***^so ^*showed similar performance to **F***^mp^*, but it filtered out more potential neighbors (288 for  versus 32 for ) and removed slightly fewer non-neighbors (39.1% for  versus 40.4% for ). The **F***^an ^*filters showed the least loss of potential neighbors (4 for  versus 32 for ), but also removed the least non-neighbors (29.0% for  versus 40.4% for ).

**Figure 10:**
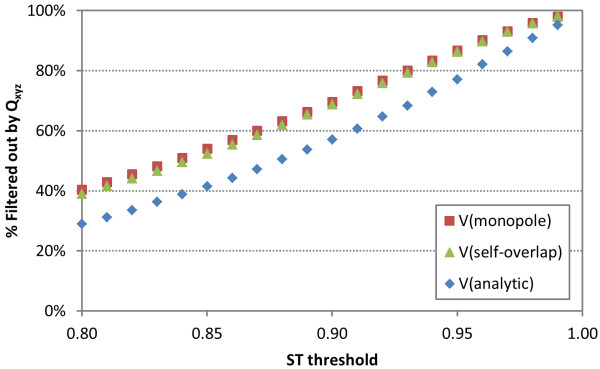
**Shape compatibility filtering efficiency**. Performance of the **F*****_xyz _***quadrupole filter to filter conformer pairs at different ST threshold values.

Effects of the ST threshold for PubChem 3-D neighboring upon the efficiency of the quadrupole filters were also investigated by generating a set of quadrupole filters, each using a different ST threshold, ranging from 0.80 to 0.99 with an increment of 0.01. As shown in Figure 10, the fraction of molecule pairs filtered increases almost linearly as a function of the ST threshold. For the entire ST range tested, the  and  filters showed better efficiencies than the  filter.

### 3. Application of 3-D neighbor filters using quadrupole moment differences

Given that filtering conformer pairs using steric shape quadrupoles is effective with minimal loss of potential 3-D neighbors, a "real world" test is made with  to see how use of these filters in the context of PubChem 3-D neighboring improves throughput. To achieve this, comparison is made to earlier benchmarks [7] whereby a set of known drugs and other molecules of keen biomedical interest are neighbored against the 3-D contents of PubChem. Table 4 and Table 5 summarize the results of these tests.

**Table 4:** Acceleration of PubChem 3-D neighboring using the  quadrupole filter

	Diverse Conformer Count				
	
	1	3	5	7	10
Average *Query *Conformers per compound	1.0	2.7	4.1	5.4	7.2
Average *Search *Conformers per compound	1.0	3.0	4.9	6.7	9.4
Total Compound Pairs (billions)	110	110	110	110	110
Total Conformer Pairs (billions)	110	866	2,200	4,010	7,510
Total Search Time before (days)	8.2	68.5	163.1	296.9	564.9
Total Search Time after (days)	6.5	55.3	118.5	224.4	410.9
Conformer pair throughput (thousands/sec)	196	181	215	207	212
% Speed up	26%	24%	38%	32%	38%

Change in throughput as a result of quadrupole filtering when generating 3-D neighbors as a function of diverse conformer count for the 4,218 chemical structures (*Query*) with known biological function when neighbored against the entire "live" PubChem3D contents (*Search*) of 26,157,365 small molecules.

**Table 5:** Efficiency of the  quadrupole filter

	Diverse Conformer Count				
	
	1	3	5	7	10
Total Conformer Pairs (billions)	110	866	2,200	4,010	7,510
CT Feature Filter	65.7%	64.8%	65.0%	64.9%	64.7%
CT Volume Filter	0.2%	0.2%	0.2%	0.2%	0.2%
ST Volume Filter	2.9%	2.7%	2.6%	2.5%	2.4%
Filter	6.3%	6.1%	5.9%	5.9%	5.9%
Filter	0.7%	0.7%	0.7%	0.7%	0.7%
Filter	0.4%	0.4%	0.4%	0.4%	0.4%
Alignment Recycling Fingerprint	2.0%	2.2%	2.2%	2.2%	2.3%
Alignment Recycling Overlap	21.5%	22.8%	22.8%	23.1%	23.3%
					
Filtering Time	1.3%	1.2%	1.2%	1.2%	1.1%
Alignment Recycling Time	75.8%	84.1%	86.1%	87.3%	88.9%
Superposition Optimization Time	12.4%	9.3%	8.5%	7.8%	7.0%
Other Overhead Time	10.5%	5.4%	4.2%	3.7%	3.1%

Relative performance of 3-D neighboring upon the addition of quadrupole filtering as a function of diverse conformer count for the 4,218 chemical structures (*Query*) with known biological function when neighbored against the entire "live" PubChem3D contents (*Search*) of 26,157,365 small molecules.

Considering PubChem 3-D neighboring is a precomputed similarity search, one can see that the neighboring throughput improvements using  are substantial, with an average improvement of 31% across the range of conformer counts per compound. Perhaps surprising is that the  filtering removes only 7% of the conformer pairs, yet achieves a 31% neighboring throughput improvement. This emphasizes the dramatic cost/benefit difference between the computation necessary to achieve the 7% reduction versus what is expended in its absence.

It is important to note that  is not the first filter applied in Table 5, meaning that there are three other filters utilized before . The filter ordering is such so as to maximize the cost/benefit of each filter. To examine what happens if  is used as the first filter, neighboring is repeated for the case of one diverse conformer per compound. When used first,  removes 48.3% of all conformer pairs (44.8%, 2.1%, and 1.4% conformer pairs for , , and , applied in that order, respectively) versus the 7.4% as shown in Table 5. The CT Feature, CT Volume, and ST Volume filters, applied in that order, remove 27.9%, 0.1%, and 0.002% conformer pairs, respectively, when  is applied first.

## Conclusion

Simple molecular shape descriptors, volume and steric quadrupole moments (embodying the length, width, and height of a shape), of 4.18 billion 3-D neighbor pairs resulting from PubChem 3-D neighboring of 17.1 million single conformer molecules were analyzed. The maximum quadrupole differences between neighbor conformers were determined. This examination demonstrated a distinct dependency of shape similarity upon quadrupole variation. With some slight modification of fringe regions, the results of this analysis were turned into computationally inexpensive, yet highly effective set of filters capable of removing 3-D conformer pairs that cannot meet a required shape similarity, using only knowledge of the volume and steric quadrupole moments of the conformer pair. When applied in the context of shape similarity searching, these filters can significantly improve throughput performance by avoiding expensive superposition optimization computation of conformer pairs that cannot possibly meet a pre-defined shape similarity search threshold.

The filters devised were tested using a dataset of 13.2 billion compound pairs. The quadrupole filters based on a monopole volume showed the best efficacy, while the filters using an analytic volume had the lowest efficacy. For all the three volume types, the *Q_x _*filters eliminated a larger portion of the compound pairs than the *Q_y _*and *Q_z _*filters. When the filters were used in a series simultaneously, they could eliminate 30~40% of non-neighbor pairs, with the removal of a negligible amount of potential neighbors. For example, the *Q_xyz _*filter based on the monopole volumes () could eliminate 40.4% of the 13.2 billion compound pairs with a loss of 32 potential neighbors out of 24 million at a shape Tanimoto (ST) threshold of 0.80. It was also demonstrated that this filtering efficiency improves linearly as a function of shape similarity threshold approaching 100% efficiency at an ST threshold of 0.99. Further testing of the  filters in the context of PubChem 3-D neighboring processing resulted in conformer pair throughput improvements of 31% on average.

In summary, the quadrupole filters developed in this study can speed up the PubChem 3-D neighbor processing with a negligible loss of the 3-D neighbors. However, its applicability is not just limited to PubChem 3-D neighboring. The results of the present study also suggest that the shape multipole moments can be applied generally to enhance the speed of 3-D similarity search methods by the rapid preclusion of dissimilar molecules that cannot be a result. This approach may be able to significantly speed up 3-D similarity search, especially if the 3-D shape superposition optimization is a bottleneck of the similarity search.

## Materials and methods

### 1. Datasets

At the time of project initiation, PubChem 3-D neighboring of 17,143,181 unique molecules (ranging from CID 1 to CID 25,000,000) had been completed using a single conformer per compound, yielding 4,182,412,802 3-D neighbors. Using the Shape Toolkit from the OpenEye Software [19], the analytic volume (*V_an_*), monopole volume (*V_mp_*), self-overlap volume (*V_so_*), and steric shape quadrupole moments (*Q_x_, Q_y_*, and *Q_z_*) were computed for the theoretical conformer of all 17.1 million molecules. See Figures 2 and 4 for the distributions of the computed values.

### 2. Filter generation

The quadrupole filters developed for pre-screening conformer-pairs based on quadrupole differences as a function of shape similarity ST threshold were generated using the following steps:

1) The 4.18 billion 3-D neighbor pairs and their associated data were obtained from PubChem.

2) The volumes (*V_mp_, V_so_*, and *V_an_*) and quadrupole components (*Q_x_, Q_y_*, and *Q_z_*) of the compound pair for each 3-D neighbor were converted into integers using **Equations 4 **and **5 **to yield , , , , , and , respectively. The denominator *BinSize *was 5.0 Å^3 ^for all three volume types and 2.5 Å^5^, 0.5 Å^5^, and 0.1 Å^5^, for *Q_x_, Q_y_*, and *Q_z_*, respectively.

3) For each of the three binned volume types, the following was performed using the 3-D neighbor pairs (in this case using  as an example):

a) Of the two conformers in a 3-D neighbor, the one with the smaller  value was designated as molecule 1 and the other as molecule 2. When the  value was the same for both, the one with the smaller  value was designated as molecule 1. If both the  and  values were the same for both, the one with the smaller  was designated as molecule 1. If  was also the same for both molecules, the one with the smaller  was designated as molecule 2. If all four descriptors are the same for both molecules, the one that appears first for the pair was designated as molecule 1.

b) For each of the three binned quadrupole components, and using  as an example:

i) 3-D neighbors were binned according to three indices, , , and , where subscripts 1 and 2 indicate molecules 1 and 2, respectively, determined in step 3a, and  is the *Q_x _*difference between the two molecules.

ii) The neighbor count for all (, , ) bins was analyzed to find the maximum possible absolute value of  for a given (, ) pair. It results in the  difference maps as a function of binned volume pairs [see panels (a) and (b) in Figures 7, 8 and 9].

iii) The  difference maps were modified, as described in the next section, to generate a final *Q_x _*filter based on monopole volumes () [see panels (c) and (d) in Figures 7, 8 and 9].

4) To obtain filters effective at an ST threshold other than ≥ 0.80, first restrict the original 4.18 billion 3-D neighbor pairs to those at or above the desired ST threshold and repeat step 3.

### 3. Modification of filters

**Figure 11:**
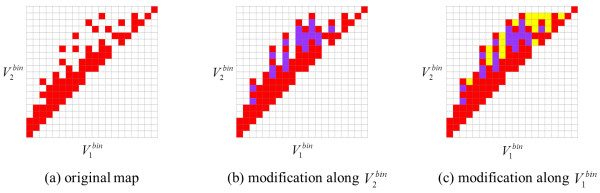
**Transformation of shape compatibility map into a filter**. Schematic diagram describing modification of an original difference map at a given Δ*Q^bin ^*value: (a) in an original map, neighbor regions are indicated in red, (b) all empty bins between the minimum and maximum values of  at each  were set to neighbor regions (purple), and (c) all empty bins between the minimum and maximum values of  at each  (yellow) were set to neighbor regions.

Figure 11 shows a schematic diagram describing how an original difference map is modified at a given Δ*Q^bin ^*value. In an original map [panel (a) of Figure 11], the (, ) bins that have population are indicated in red. Note that not all bins are populated between the minimum and maximum values of  for a given  in the fringe area. It is likely that these bins could be occupied by 3-D neighbors in the future, simply lacking an example at this time. Therefore, these bins are included in the neighbor regions [as shown in panel (b) of Figure 11] at the given Δ*Q^bin ^*value. Similarly, any empty bins within the range of  at a given  are also set in the neighbor regions [panel (c) of Figure 11] for the given Δ*Q^bin ^*value.

**Figure 12:**
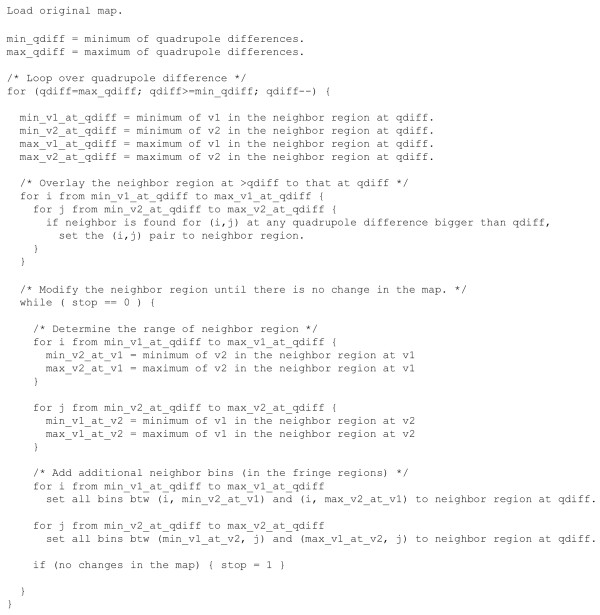
**Pseudo code to transform shape compatibility map into a filter**.

This procedure is performed for all unique Δ*Q^bin ^*values starting with the maximum. As lesser Δ*Q^bin ^*values are considered in this correction, greater Δ*Q^bin ^*values are considered at the Δ*Q^bin ^*value being considered. A pseudo-code implementation of this procedure is shown in Figure 12. All quadrupole filters resulting from this modification are available in Additional file 1.

### 4. Efficiency test of filters

To test the efficiency of the quadrupole filters devised, two sets of molecules were chosen. One set contains molecules in the PubChem CID range of 1 ~ 25,000,000, and the other contains those in the CID range of 25,000,001~25,001,000. Because a theoretical conformer was not generated for all CIDs or because compound records were not "live", the two datasets had 17,488,897 and 753 molecules, respectively. All-by-all comparison between the two sets gives 13,169,139,441 CID pairs. Using the first diverse conformer for each compound, the ST values for these 13.2 billion pairs were computed using ROCS [20] from OpenEye software, Inc., consuming ~419 CPU days in total, and stored. These ST scores were used to estimate how many CID pairs would be filtered out when applying the quadrupole filters as a function of volume type and as a function of ST threshold, for example, as demonstrated in Table 3 and Figure 10.

### 5. Effect of Quadrupole filters on PubChem3D Neighboring

One aspect of this effort is to examine the change in real-world efficiency of PubChem3D neighboring processing when using quadrupole filters while computing the 3-D "Similar Conformers" relationship. To achieve this, the set of 4,218 biologically relevant chemical structures with known pharmacological actions from our earlier efforts [7] was used. These small molecules with known biological action (*Query set*) were neighbored against 26,157,365 compound records (*Search set*), representing the entire "live" PubChem3D contents as of Oct. 2010, using up to 1, 3, 5, 7, and 10 diverse conformers per compound for both compound sets. Timing and efficiency differences with our earlier work are given in Tables 4 and 5.

## Competing interests

The authors declare that they have no competing interests.

## Authors' contributions

SK analyzed the quadrupole differences of the 3-D neighbors, generated the quadrupole filters, and wrote the first draft. EEB supervised the project and revised manuscript. SHB reviewed the final manuscript. All authors read and approved the final manuscript.

## Supplementary Material

Additional file 1 **Quadrupole filters**. A zip archive of text files containing information on the maximum quadrupole differences as a function of molecular volumes. Click here for file
